# Immune Monitoring upon Treatment with Biologics in Sjögren’s Syndrome: The What, Where, When, and How

**DOI:** 10.3390/biom11010116

**Published:** 2021-01-16

**Authors:** Joyce J.B.C. van Beers, Jan G.M.C. Damoiseaux

**Affiliations:** 1 Central Diagnostic Laboratory Maastricht University Medical Center, Laboratory Specialist in Medical Immunology and Clinical Chemistry, 6202 AZ Maastricht, The Netherlands; 2 Central Diagnostic Laboratory Maastricht University Medical Center, Laboratory Specialist in Medical Immunology, 6202 AZ Maastricht, The Netherlands; jan.damoiseaux@mumc.nl

**Keywords:** immune monitoring, biologics, Sjögren’s syndrome, Sjögren’s disease

## Abstract

Over the years, a wide variety of therapeutic antibodies has been successfully introduced in the auto-immunology clinic, and many more are on the way. Many of these treatments address either a pathogenic circulating molecule or a cell-bound molecule. Whereas addressing the former target results in neutralization of the soluble factor and binding to the latter target either inhibits cellular function or induces selective cell death. If this targeted molecule or cell is part of the immune system, this therapy evokes a state of immunodeficiency with infections as a possible consequence. Therefore, immune monitoring is needed to prevent such adverse side effects of immunotherapy. In this paper, different immunotherapies used in Sjögren’s syndrome, as well as different approaches to monitoring the immune system, are discussed.

## 1. Primary Sjögren’s Syndrome

Primary Sjögren’s syndrome (pSS) is a systemic, chronic autoimmune disease mainly affecting the exocrine glands of the body such as the lacrimal and salivary glands. The symptoms of pSS can vary from sicca symptoms (dryness of the eyes, oral cavity, pharynx, larynx, and/or vagina), more general symptoms (fatigue, chronic pain, depression, and anxiety), to systemic or extra-glandular symptoms (e.g., lymphoma, arthritis, interstitial lung disease, and renal failure) [1]. Besides pSS, SS can also occur secondary (sSS) to another autoimmune disease (e.g., systemic lupus erythematosus, rheumatoid arthritis).

The estimated incidence of pSS is 4 per 1000 patients per year with an estimated overall prevalence between 0.1% and 4.8% in Europe. This is probably an underestimate because some symptoms are not specific to pSS and the disease is very heterogeneous [1].

The diagnosis of pSS is most often based upon the 2016 classification criteria of the American College of Rheumatology (ACR)/European League against rheumatism (EULAR) (Table 1) [2]. Systemic disease activity can be evaluated with EULAR Sjögren’s syndrome disease activity index (ESSDAI) [3]. The ESSDAI score includes different domains (e.g., organs involved) to determine disease activity and is designed to assess the systemic activity of patients with pSS [4,5]. In addition, the EULAR SS Patient Reported Index (ESSPRI) is designed to assess symptoms with the help of a questionnaire [5,6].

Lymphocytic (B- as well as T-cell) infiltrations of exocrine glands and (systemic) hyperactivation of B-cells are characteristics seen in patients with pSS [7,8]. Lymphocytic infiltrations can also occur beyond the exocrine glands. As a result of lymphocyte infiltration, interstitial nephritis, autoimmune primary biliary cholangitis, and obstructive bronchiolitis can occur. Moreover, B-cell hyperactivation can lead to immune depositions (mainly due to cryoglobulinemia), which in turn can lead to palpable purpura, glomerulonephritis, interstitial pneumonitis, interstitial lung disease, and peripheral neuropathy. Finally, patients with pSS have 15–20 times more risk of developing B-cell non-Hodgkin lymphoma (B-cell NHL), mainly lymphoma of mucosa-associated lymphoid tissue (MALT), compared to healthy individuals [9].

Immunological markers can play a role in pSS diagnosis. The main markers are autoantibodies (mainly anti-SSA/Ro antibodies, but also other autoantibodies such as (IgA) rheumatoid factor (RF) and anti-SSB/La are often present), cryoglobulin (associated with lymphoma), and low complement levels [7,10,11].

**Figure 1:**
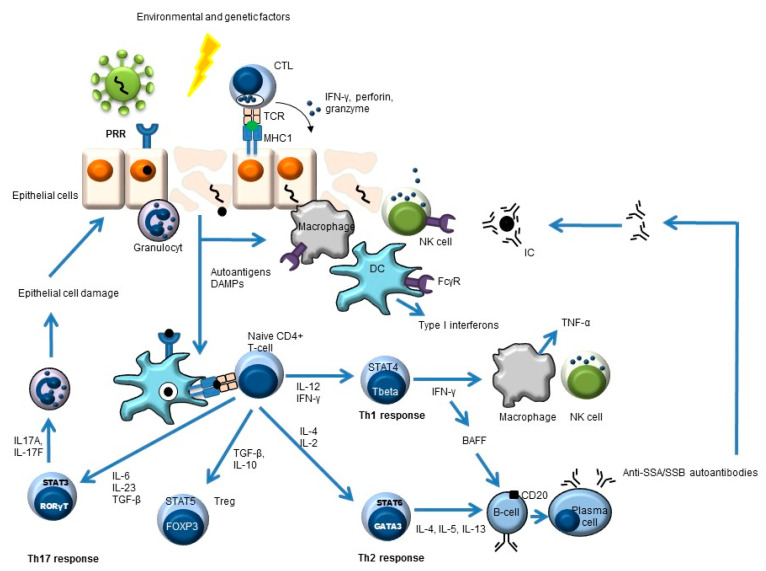
Simplified overview of the key players in pSS pathogenesis. Environmental and genetic factors may lead to pSS. An example of an environmental trigger may be a virus. This leads to antigen uptake by antigen-presenting cells (e.g., dendritic cells, DC) and subsequent antigen presentation to CD4-positive naïve T-cells. These naïve cells will develop (cytokine-dependent) into different T-helper (Th) cells or into regulatory T-cells (Treg) with different effector functions. The Th2 response can lead to the formation of autoantibodies (e.g., anti-SSA, anti-SSB) and, subsequently, to immune complex (IC) formation.

Besides a critical role for B-cells in the pathogenesis of pSS, other cells also play an important role, such as stromal and epithelial cells, cells of the innate immune system (e.g., dendritic cells, monocytes/macrophages), and T-cells (e.g., Th1, Th2, Th17, and follicular Th cells) (Figure 1) [7,12,13,14,15,16].

## 2. Targets of Biologics in pSS—The “What”

SS is a disease difficult to manage. To date, no cure is available. The main goals for treatment are relief of symptoms and prevention of complications. Artificial tears, lubricants, and saliva substitutes are used to reduce sicca symptoms. Many patients with pSS use a muscarinic receptor agonist (such as pilocarpine), which stimulates residual salivary gland function [10,17,18].

Non-steroidal anti-inflammatory drugs (NSAIDs) are often used to target more general symptoms. Other agents used in pSS are hydroxychloroquine (for arthralgia, low–moderate arthritis, and cutaneous involvement) and methotrexate (for more severe arthritis and when hydroxychloroquine is not sufficient), although there is little evidence for their efficacy in pSS.

In the case of severe organ manifestations, patients are often treated with immunosuppressive agents, including glucocorticoids, methotrexate, mycophenolate sodium, azathioprine, and cyclophosphamide, in accordance with available guidelines regarding connective tissue diseases [10,17].

**Figure 2:**
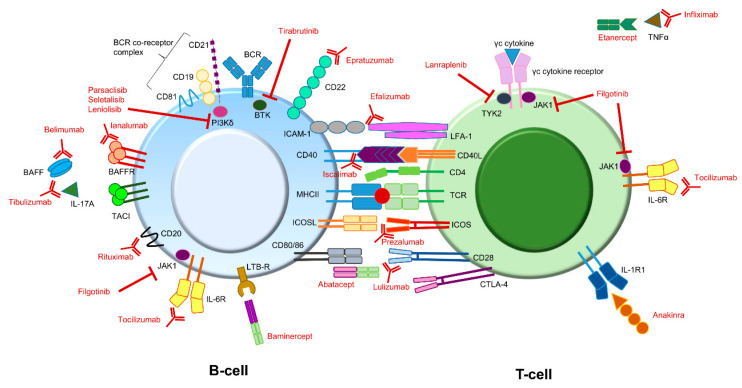
Potential immune therapy targets for treating pSS symptoms. Compounds (depicted in red) directed against different targets involved in B-cell or T-cell signaling have already been addressed or are currently being addressed in different studies. Some interactions can lead to different effector functions. For instance, BAFF can bind to the BAFFR as well as to TACI. In addition, CD80/86 molecules on B-cells can interact with both CD28 as well as CTLA-4 on T-cells. BAFFR and CD28 binding both lead to an activating signal, while binding to TACI and CTLA-4 both lead to an inhibiting signal. Note: The above figure is a simplified version. It does not depict all potential targets present on or in B- and T-cells. In addition, some target molecules are also present on other immune cells (e.g., IL-1R). BAFF: B-cell activating factor; BCR: B-cell receptor; BTK: Bruton’s tyrosine kinase; CTLA-4: cytotoxic T-lymphocyte antigen 4; ICAM-1: intercellular adhesion molecule 1; ICOS: Inducible T-cell Co-stimulator; ICOSL: Inducible T-cell Co-stimulator ligand; IL: interleukin; JAK1: Janus kinase 1; LFA-1: lymphocyte function-associated antigen 1; LTB-R: lymphotoxin beta receptor; MHC: major histocompatibility complex (also known as HLA, human leukocyte antigen); TACI: transmembrane activator and calcium modulator and cyclophilin ligand (CAML) interactor; TCR: T-cell receptor; TNF tumor necrosis factor; TYK2: tyrosine kinase 2.

Besides the more conventional therapy, for which the efficacy is (for the most part) not evidence-based, the efficacy of immune therapy targeting different molecules is currently under investigation as an option for treating pSS symptoms (Figure 2) [19,20].

The presence of autoantibodies, hypergammaglobulinemia, and the increased risk of B-cell NHL development emphasize the important role of B-cells in pSS pathogenesis [7]. One of the most studied B-cell therapies in pSS involves rituximab. Rituximab is a chimeric monoclonal antibody that targets the CD20 molecule expressed by most B-cells. The binding of CD20 by monoclonal antibodies can lead to apoptosis or cellular cytotoxicity, either by complement-dependent (CDC) or antibody-dependent (ADCC) mechanisms [21,22]. Many studies have shown the efficacy of rituximab in at least one of the systemic outcomes analyzed (global response, organ-specific response, ESSDAI reduction, and/or glucocorticoid reduction). However, studies have also reported on the lack of clinical efficacy and cost-effectivity [18,23,24]. Although efficacy is partly lacking, treatment guidelines for pSS validate the use of rituximab in the management of selected cases of pSS [18].

Belimumab is a monoclonal antibody that targets the TNF cytokine family member B-cell activating factor (BAFF), also known as B-cell lymphocyte stimulator (BLyS). BAFF plays an important role in B-cell maturation, survival, antibody production, and lymphoproliferation. BAFF is primarily induced by type I and type II interferons and is thought to play a central role in pSS pathogenesis. Increased levels of BAFF are found in saliva, sera, and affected tissue of pSS patients compared to healthy individuals [21,25]. Little is known so far about the efficacy of belimumab in pSS. The few studies that have been published show that belimumab seems to be promising in reducing systemic activity, gland enlargement, lymphadenopathies, articular manifestation, and B cell biomarkers (i.e., immunoglobulin and RF levels) [26,27].

BAFF binds to the BAFF receptor (BAFF-R; CD268) or to the transmembrane activator and calcium-modulating cyclophilin ligand interactor (TACI; CD267). In B-cells, signaling via BAFF-R leads to B-cell survival, while signaling via TACI leads to negative regulation of B-cell proliferation. In addition, BAFF-R and TACI both play a role in T-cell-independent antibody class-switching induction [28]. Anti-BAFF-R therapy (ianalumab) eliminates BAFF-R-positive immature and mature B-cells through ADCC [29]. The efficacy of ianalumab, which prevents BAFF signaling, was recently addressed in pSS. A positive therapeutic effect (improvement in ESSDAI as well as ESSPRI scores and reduction in serum Ig light chains) was observed suggesting that ianalumab could provide therapeutic benefits without major side effects [29].

Epratuzumab targets CD22, a co-receptor of the B-cell receptor. Epratuzumab does not lead to complete B-cell depletion, but it has immunomodulatory effects. Small studies with epratuzumab showed potential clinical benefits. However, this still needs to be confirmed by randomized controlled trials [25,30].

Lymphotoxin-β is involved in ectopic lymphoid structure organization, in particular germinal center (GC) formation. However, trials with baminercept, a lymphotoxin-beta receptor (LTB-R) fusion protein, failed. No differences were observed compared to the control group [31].

Immune responses are regulated via co-stimulatory and co-inhibitory signals. In auto-immune diseases, these signals are often impaired [32]. The TCR of CD4+ T-cells (Th-cells) recognizes antigens presented by antigen-presenting cells (APC), such as the B-cell, via human leukocyte antigen class 2 molecules (HLA-II). For activation T-cells require co-stimulatory signals. Major costimulatory pathways in pSS include CD28-CD80/86, ICOS-ICOS-L, CD40L-CD40, and LFA-1-ICAM interactions [33].

CD28 and CTLA-4 (CD152) both bind the same ligands (CD80 and CD86). CD28 provides a stimulating/activating signal, and CTLA-4 provides an inhibitory signal. Abatacept is a soluble fusion protein, consisting of the extracellular domain of CTLA-4 and a fragment of the Fc portion of human IgG1 (CTLA-a-Ig). Abatacept binds stronger to CD80/CD86 (B7 molecules) than CD28, leading to the prevention of T-cell activation [34,35]. The results from clinical trials with abatacept are not consistent. Some studies showed no decrease in disease activity (using the ESSDAI score), while others showed a reduction in disease activity and also a reduction in cytokine and autoantibody levels [35,36,37]. A study in which CD28 was the target of monoclonal antibody therapy (lulizumab) was terminated before it was completed. The reason for this remains unknown [38,39].

Besides CD28, ICOS (CD278) is also upregulated in T-cells in pSS patients. ICOS is specifically involved in T-cell-dependent B-cell activation. ICOS–ICOS-L interactions ultimately lead to class-switch recombination and terminal differentiation of B-cells into memory B-cells or plasma cells [35,40]. Prezalumab is a monoclonal antibody directed against ICOS. In a randomized, placebo-controlled study, besides reduction in RF levels, no clinical improvement was detected [41]. The best-known member of the TNF/TNFR family is TNFα, which mainly plays a crucial role in autoinflammatory diseases (i.e., psoriasis, inflammatory bowel disease, spondylo-arthritis) [42]. CD40L is another member of the TNF/TNFR family. It has been shown that increased CD40L (CD154) expression is observed in pSS patients. Therefore, targeting the CD40L-CD40 pathway has been suggested [43]. Iscalimab is a monoclonal antibody directed against CD40. Initial findings of treatment with iscalimab are promising, but further studies are needed to confirm the potential clinical benefits [44].

A mouse desiccating dry eye model showed beneficial effects by blocking the interaction between LFA-1 (Lymphocyte function-associated antigen 1; CD11a) and ICAM-1 (Intercellular Adhesion Molecule 1; CD54). However, a study in humans with anti-LFA-1 (efalizumab) was terminated due to serious side effects [45].

The common γ-chain receptors (e.g., IL-2R, IL-4R, IL-7R, IL19R, IL-15R, IL-21R) bind cytokines that are important for T-cell development and differentiation. Signaling occurs via the JAK-STAT signaling pathway involving, amongst other small molecules, JAK1 and TYK2 [46]. Results from studies that target small molecules such as JAK1 (filgotinib) or TYK2 (lanraplenib) are still ongoing. In addition, small molecules (e.g., PI3kδ, BTK) involved in B-cell receptor (BCR) signaling are under investigation as potential treatment targets [33]. Iguratimod, which acts on T- as well as B-cells by inhibiting various inflammatory signaling pathways, is a promising compound for pSS treatment. The alleviation of symptoms and reduction in ESSDAI score have been reported in pSS patients currently under investigation [47,48,49].

Finally, upregulation of several pro-inflammatory cytokines (e.g., IFN, TNFα, IL-1β, IL-6, BAFF) has been detected in pSS [50]. Several clinical trials targeting anti-TNFα (by infliximab and etanercept) and anakinra (interleukine-1-receptor antagonist) failed in pSS [51,52,53]. Some case studies showed improvement of certain systemic (neurological) symptoms with tocilizumab (anti-IL-6 receptor, IL-6R, or CD126); however, no successful trials have been completed yet [54,55].

## 3. Immune Monitoring—The “Where”

Immune monitoring provides information about the status of the immune system throughout treatment. Information about the numbers and functionality of immune cells and molecules gives insight into the treatment response and potential side effects (e.g., risk of infection).

Assays for immune monitoring mainly focus on lymphocytes (e.g., absolute numbers, maturation pathways during reconstitution), immunoglobulin levels (e.g., total IgG, autoantibodies), cytokines (e.g., BAFF), and complement (e.g., in case of monitoring the effects of Eculizumab for cryoglobulinemia). There are many sources of lymphocytes, such as peripheral blood, lymph nodes, vaccine injection sites, or exocrine glands, but peripheral blood is the most widely used source for immune monitoring assays mainly because it is the easiest to obtain. The source, however, is secondary to pre-analytical procedures. Collection, processing, and storage can be critical for the interpretation of immune monitoring assays [56].

## 4. Immune Monitoring—The “When”

The main focus of immune monitoring in pSS, as described below, is on B-cell depletion therapies because most is known about these treatments from clinical studies.

Before starting treatment, it is suggested to screen patients for at least baseline immunoglobulin concentrations, memory B-cell subset (un-switched and switched), and plasmablast numbers. Delayed B-cell reconstitution, B-cell recovery failure, and/or development of hypogammaglobulinemia after treatment may be observed in individuals with low switched memory B cells and plasmablasts at baseline [21]. Ideally, if one would like to address cytokine levels (e.g., BAFF or IL-6) post-treatment, cytokines should also be measured at baseline.

Different studies showed that the half-life of rituximab varies between 18 and 23 days [21].

It has been shown that after 5 weeks of rituximab treatment in pSS all B-cells are depleted, and B-cells typically re-occur in the peripheral blood after 6 to 9 months. However, some patients may remain depleted of B-cells much longer. The time to complete B-cell reconstitution varies among patients [57].

If B-cells are still present 3 months after treatment, especially in combination with the presence of memory cells (see Section 5), a new bolus or switching to another treatment strategy should be considered [58]. If B-cells are absent, patients should be monitored at a later time-point (after a couple of months when B-cell reconstitution is expected) to address B-cell maturation and differentiation. Serum immunoglobulin levels (total IgG, IgM, and IgA) should be determined, at least, before each retreatment because long-lasting depletion of B cells may lead to decreased antibody production [59]. In addition, immunoglobulin levels (total, IgG, IgM, and IgA) should be obtained if a patient develops a serious infection or repeated infections [21].

It has been suggested that B-cell depletion is influenced by Fcγ-receptor (FcγRIIIa or CD16a) polymorphisms on effector cells [60]. FcγRIIIa binds the constant domain of the monoclonal antibody, which is the first step towards ADCC. In NK cells, FcγRIIIa is essential for ADCC. NK-cells that have an altered Fcγ-receptor expression, or even lack expression of this receptor, could potentially be unable to perform ADCC. However, a small study that addressed B-cell repopulation after rituximab treatment in pSS did not observe an association between FcγRIIIa polymorphism and treatment effects [61]. FcγRIIIa genotyping is therefore not necessary for immune monitoring in pSS.

Because rituximab is a chimeric monoclonal antibody, antibodies against rituximab may be formed. The presence of anti-rituximab antibodies can influence therapy efficacy because they can neutralize rituximab and prevent B-cell depletion. Patients with anti-rituximab antibodies can benefit from humanized anti-CD20 therapy [62].

## 5. Immune Monitoring—The “How”

**Figure 3:**
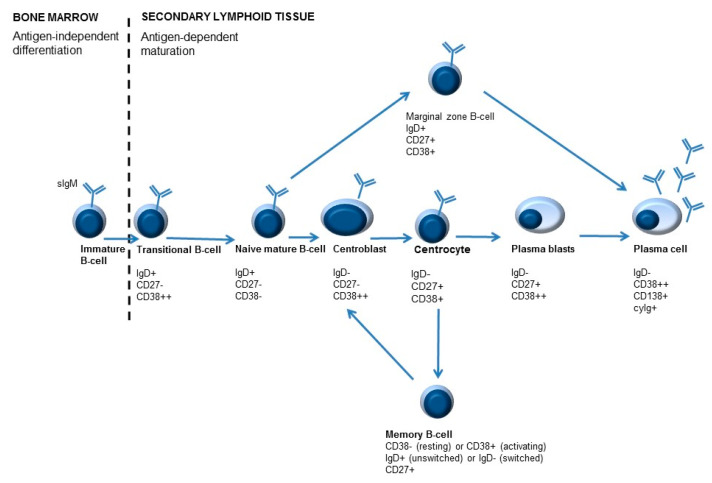
Simplified overview of B-cell maturation. B-cell development starts in the bone marrow. During development different B-cell maturation stages are identified, characterized by specific molecules on their surface. After B-cells leave the bone marrow, maturation and differentiation may occur. Differentiation of B-cells is antigen-dependent. Antigen recognition occurs in the lymphoid follicles, where centroblasts develop into centrocytes in the so-called germinal center (GC) reaction. As a result of B-cell activation, immunoglobulins are ultimately secreted by plasma cells. An important feature of adaptive immunity is memory formation, which can lead to a quicker and stronger response when the immune system encounters the same antigen for the second time. Memory cells can be divided into unswitched memory B-cells or switched memory B-cells, depending on IgD expression (positive or negative, respectively).

Numbers of lymphocyte subsets are routinely addressed by immunophenotyping with flow cytometry. The majority of B-cells found in the peripheral blood are naive B-cells, but also other B-cell subpopulations can be detected [63]. The different B-cell subpopulations can be identified in the blood with the help of sIgD, CD27, and CD38 markers, making it possible to monitor B-cell maturation and differentiation (Figure 3). Adding surface immunoglobulin (sIg) IgM (sIgM), IgA (sIgA), or IgG (sIgG) can provide additional information about isotype-switched subsets with different functions and, potentially, different roles in autoimmunity [58].

B-cells that are newly emigrated from the bone marrow to the secondary lymphoid organs are called transitional B-cells (IgD+/CD27−/CD38++ or T1 B-cells). These cells can ultimately either develop into marginal zone B-lymphocytes (IgD+/CD27+/CD38+) or centroblasts (sIgD−/CD27+/CD38++) [61]. Mature naive B-cells (IgD+/CD27−/CD38− and IgD+/CD27−/CD38+ or Bm1 and Bm2) can become activated (T-cell dependent activation) in secondary lymphoid tissues. These B-cells can mature into centroblasts (IgD−/CD27+/CD38++ or Bm3). After the process of somatic hypermutation, centrocytes are formed (CD38+/IgD− or Bm4). These B-cells can either differentiate to memory B-cells (IgD+/−/CD27+/CD38+/− or Bm5) and, depending on class switch recombination, into switched memory B-cells (CD27+/IgD−/CD38−) or to plasmablasts (IgD−/CD27+/CD38++). Plasmablasts can ultimately develop into plasma cells (CD38+/CD138+/cyIg) [7,61,64].

After rituximab (or anti-CD20 therapy in general), transitional B-cells (IgD+/CD27−/CD38++) are the first B-cell to re-occur in the peripheral blood. Reduced numbers of memory B-cells after rituximab treatment have been identified as a reliable indicator of disease course. The number of memory B-cells remains to be low for approximately one year after treatment (this can vary between patients) and gradually increases from that point [65]. Increased numbers of memory B-cells shortly after treatment have been suggested to be a marker of relapse or therapy resistance [66].

Rituximab only depletes CD20-positive B-cells. Precursor B-cells and plasmablasts/plasma cells are spared. In contrast, belimumab has rapid effects on naïve B cells and B cells of earlier developmental stages, while B cells of later stages, such as plasmablasts, show delayed or no responses [67].

Besides B-cells, T-cells should also be monitored after B-cell-directed treatment. Immune monitoring mainly aims at the different CD4− and CD8− positive T-cell subsets (naive and memory), but also regulatory T-cells (Treg) and follicular T-cells (Tfh) can be addressed. Most Tfh cells are present in germinal centers (GC), where they play an important role in activating germinal center B cells. Tfh cells are considered to be the most potent T-cells that induce hyperactivation of B-cells. This will ultimately lead to immunoglobulin isotype switching and the production of long-lasting plasma cells. It has been shown that the frequency of Tfh cells is correlated with lymphocytic infiltration, the number of B cells, elevated serum and salivary gland Ig levels, and autoimmunity [68,69]. Tfh cells can also be found in the peripheral circulation and can be identified by CXCR5 expression on CD4 T-cells with flow cytometry [13].

Measuring the levels of cytokines in peripheral blood samples (without in vitro stimulation), but also in tears and saliva, could provide information about disease activity [70,71,72]. Nowadays, multiple cytokines and chemokines can be measured simultaneously in multiplex assays. Increased cytokine levels, such as IL-6 and BAFF, have been found in pSS patients. An association between elevated BAFF levels and a shorter delay in B-cell reconstitution after rituximab has been observed [61]. Interpretation of cytokine levels is often difficult due to the lack of baseline and reference values.

For many years immune monitoring mainly relied on flow cytometry. In flow cytometry, antibodies with a fluorescent tag are used. A drawback of this technique is the limited number of fluorescent tags that can be used in a single tube mainly due to spillover between different fluorescent spectra.

New technical approaches in molecular analyses have created the opportunity for the application of microarray and sequencing technologies to provide a comprehensive view of the immune response at the transcriptional level [73]. In addition, mass spectrometry is used as a novel tool in immune monitoring [73]. Cytometry by time-of-flight (cyTOF) uses multiple antibodies tagged with multiple copies of heavy metal ions [73,74,75]. With mass spectrometry (e.g., cyTOF), it is possible to identify more molecular features in a single assay and to reduce signal overlap and background signals [67,74,76].

## 6. Discussion

Immune monitoring is important when using biologicals addressing components of the immune system. It can, depending on the time-point and clinical question, contribute to personalized medicine by increasing therapeutic efficacy and preventing complications, thereby saving costs and potentially increasing the quality of life of patients undergoing such therapy.

Immune monitoring can be useful to predict if patients will respond to therapy. In the case of anti-CD20 therapy, plasmablasts/plasma cells are not affected by anti-CD20 therapy. The presence of increased plasmablasts could be a restriction for effectively using anti-CD20 therapy (e.g., rituximab) because the effect of B-cell depletion by CD20 therapy on immunoglobulin levels is limited. Indeed, plasma cells are not depleted because they lack the CD20 antigen. However, in the case of auto-immune diseases, immunoglobulins may be produced by short-lived plasma cells that require constant renewal to maintain immunoglobulin levels. Moreover, upon long-term treatment with B-cell-depleting therapy, long-lived plasma cells may also be affected, eventually resulting in declining immunoglobulin levels [77,78].

Immune monitoring can be useful to understand why certain patients do not show the predicted therapy response [79]. Antibodies against, for instance, rituximab can lead to rituximab neutralization and subsequently prevent B-cell depletion [62]. Moreover, CD16 deficiency can potentially interfere with the action of rituximab by preventing ADCC [60]. In addition, therapy failure can also occur when B-cells are not the key players in the pathogenesis or when B-cells are not accessible for therapy. B-cell therapy often leads to B-cell depletion in the peripheral blood; however, B-cells present in secondary organs (e.g., lymph nodes, spleen) are often not immediately (e.g., after a single dose) depleted.

Immune monitoring is useful to evaluate B-cell reconstitution. In the case of retreatment, individuals with long-term B-cell depletion should be closely monitored by addressing B-cell numbers, B-cell subsets, and immunoglobulin levels [59]. Transitional B-cells are the first B-cell to re-occur in the peripheral blood after B-cell depletion therapy [61]. As already mentioned, long-lived plasma cells are not affected by anti-CD20 therapy; therefore, immunoglobulin levels are usually not immediately affected by rituximab treatment. Effects on immunoglobulin levels, however, have been found after repeated B-cell depletion therapy and in the case of immunoglobulins produced by short-lived plasma cells [77,78].

Finally, immune monitoring is useful to monitor potential infection risk. Infections (e.g., upper respiratory tract infections, nasopharyngitis, urinary tract infections, bronchitis, sinusitis, hepatitis B reactivation) have been described in patients using rituximab [80,81]. The seriousness of the complications depends on the patient population (patients suffering from a hematological malignancy versus patients suffering from an autoimmune disorder), duration of treatment, and rituximab mono-therapy or combination therapy [80]. To protect patients from obtaining infections, patients can be vaccinated, and the efficacy of the vaccination can be monitored by measuring pre-and post-vaccination responses (e.g., specific immunoglobulin titers). Vaccination against Streptococcus pneumonia and Haemophilus influenzae type b (Hib) is common. In the case of patients with rheumatoid arthritis who received rituximab, several studies have shown a compromised antibody response after vaccination [82,83,84]. However, besides the antibody-mediated response, the cellular immune response is important in fighting infections. These findings may have implications on the timing of vaccinations and the type of vaccine used (e.g., polysaccharide vaccine versus conjugate vaccine). With an adequate vaccination scheme (e.g., at least six months after rituximab) a sufficient vaccination response can still be achieved [84], but this is obviously dependent on confirmation of the expected recovery of the B-cell compartment.

The main challenges of immune monitoring are obtaining baseline (pre-treatment) levels (mainly with respect to B-cell numbers, B-cell subsets, and immunoglobulin levels), analytical sensitivity, and standardization between different laboratories. Different laboratories use different markers to address B-cell subsets. In addition, differences exist in which and how B-cell subsets are reported. Tools and protocols provided by the EuroFlow consortium could provide a more standardized workflow [85,86].

Suggestions for immune monitoring in clinical practice for pSS are summarized in Table 2.

## 7. Conclusions

Not much is known about immune monitoring for the treatment with biologics in Sjögren’s syndrome. In this paper, we have discussed the different immunotherapies used in Sjögren’s syndrome, and we have shown the different assays currently available to monitor the immune system. As discussed, further research and harmonization are needed to optimize immune monitoring in individual patients.

## Figures and Tables

**Table 1:** The 2016 American College of Rheumatology (ACR)/European League against rheumatism (EULAR) classification criteria for primary Sjögren’s syndrome (pSS).

Item	Weight/Score	Rules for Classification
1. Labial salivary gland with focal lymphocytic sialadenitis and focus score of ≥ 1 foci/4 mm^2^	3	Applies to any individual
2. Anti-SSA/Ro-positive	3	—who meets the inclusion criteria (presence of ocular and/or oral dryness) with at least one symptom of ocular or oral dryness or ESSDAI ≥ 1
3. Ocular Staining Score ≥ 5 (or van Bijsterveld score ≥ 4) in at least one eye	1	—does not have any of the conditions listed as exclusion criteria ^a^
4. Schirmer’s test ≤ 5 mm/5 min in at least one eye	1	—and has a score of ≥ 4 when the weights from the 5 criteria items are summed
5. Unstimulated whole saliva flow rate ≤ 0.1 mL/min	1	

^a^ Exclusion criteria include history of head and neck radiation treatment, active hepatitis C infection (with confirmation by PCR), AIDS, sarcoidosis, amyloidosis, graft-versus-host disease, and IgG4-related disease [2].

**Table 2:** Suggestions for immune monitoring in pSS.

Timepoint	Suggested Assay
Baseline ^a^	IgG, IgM, IgA concentrations, B-cell numbers including memory B-cell subsets (un-switched and switched; IgD+/CD27+/CD38- and IgD-/CD27+/CD38-, respectively) and plasmablast (IgD-/CD27+/CD38++) numbers. Optional (depending on therapy): cytokine concentrations ^b^
3 months after therapy onset ^c^	B-cell numbers including memory B-cell subsets (un-switched and switched; IgD+/CD27+/CD38- and IgD-/CD27+/CD38-, respectively) and plasmablast (IgD-/CD27+/CD38++) numbers.
6–9 months after therapy onset	Lymphocyt (NK cells, T-cells and B-cells) numbers including memory B-cell subsets (un-switched and switched; IgD+/CD27+/CD38- and IgD-/CD27+/CD38-, respectively) and transitional B-cell (IgD+/CD27-/CD38++) numbers. Optional (depending on therapy): cytokine concentrations ^b^
Infections	IgG, IgM, IgA concentrations, T-cell numbers and B-cell numbers including memory B-cell subsets (un-switched and switched; IgD+/CD27+/CD38- and IgD-/CD27+/CD38-, respectively).

^a^ Before therapy onset; ^b^ If one would like to address cytokine levels (e.g., BAFF or IL-6) post-treatment, cytokines are suggested to be also measured at baseline; ^c^ If B-cells are still present, especially in combination with the presence of memory cells, a new bolus or switching to another treatment strategy should be considered.
